# Formula diet alters small intestine morphology, microbial abundance and reduces VE-cadherin and IL-10 expression in neonatal porcine model

**DOI:** 10.1186/s12876-016-0456-x

**Published:** 2016-03-22

**Authors:** Laxmi Yeruva, Nicole E. Spencer, Manish K. Saraf, Leah Hennings, Anne K. Bowlin, Mario A. Cleves, Kelly Mercer, Sree V. Chintapalli, Kartik Shankar, Roger G. Rank, Thomas M. Badger, Martin J. J. Ronis

**Affiliations:** Arkansas Children’s Nutrition Center, 15 Children’s Way, Little Rock, AR 72202 USA; Arkansas Children’s Hospital Research Institute, Little Rock, USA; Department of Pediatrics, University of Arkansas for Medical Sciences, Little Rock, USA; Department of Pathology, University of Arkansas for Medical Sciences, Little Rock, USA; Department of Pharmacology & Experimental Therapeutics, Louisiana State University Health Sciences Center, New Orleans, LA USA

**Keywords:** Nutrition, Peyer’s patches, Ileum, Jejunum, GALT

## Abstract

**Background:**

Breastfeeding is associated with a variety of positive health outcomes in children and is recommended exclusively for the first 6 months of life; however, 50–70 % of infants in the US are formula-fed. To test the hypothesis that immune system development and function in neonates and infants are significantly influenced by diet, 2-day old piglets were fed soy or milk formula (*n* = 6/group/gender) until day 21 and compared to a sow-fed group (*n* = 6/gender).

**Methods:**

Histomorphometric analyses of ileum, jejunum and Peyer’s patches were carried out, to determine the inflammation status, mRNA and protein expression of pro-inflammatory, anti-inflammatory and growth-related chemokines and cytokines.

**Results:**

In formula-fed animals, increases in ileum and jejunum villus height and crypt depth were observed in comparison to sow-fed animals (jejunum, *p* < 0.01 villus height, *p* < 0.04 crypt depth; ileum *p* < 0.001 villus height, *p* < 0.002 crypt depth). In formula-fed the lymphoid follicle size (*p* < 0.01) and germinal centers (*p* < 0.01) with in the Peyer’s patch were significantly decreased in comparison to sow-fed, indicating less immune education. In ileum, formula diet induced significant up-regulation of AMCFII, IL-8, IL-15, VEGFA, LIF, FASL, CXCL11, CCL4, CCL25 and down-regulation of IL-6, IL-9, IL-10, IL-27, IFNA4, CSF3, LOC100152038, and LOC100736831 at the transcript level. We have confirmed some of the mRNA data by measuring protein, and significant down-regulation of anti-inflammatory molecule IL-10 in comparison to sow-fed piglets was observed. To further determine the membrane protein expression in the ileum, VE-cadherin, occludin, and claudin-3, Western blot analyses were conducted. Sow fed piglets showed significantly more VE-Cadherin, which associated with levels of calcium, and putrescine measured. It is possible that differences in GI tract and immune development are related to shifts in the microbiome; notably, there were 5-fold higher amounts of Lactobacillaceae spp and 3 fold higher Clostridia spp in the sow fed group in comparison to milk formula-fed piglets, whereas in milk formula-fed pigs Enterobacteriaceae spp was 5-fold higher.

**Conclusion:**

In conclusion, formula diet alters GI morphology, microbial abundance, intestinal barrier protein VE-cadherin and anti-inflammatory molecule IL-10 expression. Further characterization of formula effects could lead to modification of infant formula to improve immune function, reduce inflammation and prevent conditions such as allergies and infections.

**Electronic supplementary material:**

The online version of this article (doi:10.1186/s12876-016-0456-x) contains supplementary material, which is available to authorized users.

## Background

Breastfeeding is associated with a variety of positive health outcomes in children and is recommended exclusively for the first 6 months of life [1]; however, 50–70 % of infants in the US are formula-fed [2]. Several studies have indicated that formula feeding has a negative impact on immediate and long-term health of children [3–6], suggesting that the complex mixture of nutrients and bioactive components in breast milk have a beneficial impact on health and disease outcomes. Many of these studies have noted increased gastrointestinal (GI) and/or upper respiratory infections, and prevalence of ear infections in formula fed infants [3–6], yet few have identified a pathophysiological basis for these outcomes. The underlying mechanisms remain to be fully elucidated, in part due to the challenges associated with specimen-collection and experimental intervention in human newborns and infants. The full implications of formula-feeding in terms of intestinal development and function, especially the GI immune system, remain to be determined, and the impact of specific components of formulas is an important consideration.

There is a paucity of data on the relative effects of breastfeeding and infant formula on gastrointestinal development and function. Studies conducted in rats and guinea pigs demonstrated that early diet influences the development of the gastrointestinal (GI) tract mucosal epithelium, gut-associated lymphoid tissue (GALT), the microbiota acquired in early life, and the mucosal immune response and tolerance. For example, in rat models of formula feeding, small intestine and colon weights were increased in comparison to suckled rats, and longer villi and deeper crypts with thicker muscle layers all along the small intestine were observed [7, 8]. In another study, piglets fed formula between postnatal days 7 and 21 showed increased wall thickness of mucosa and density (g/cm) of ileum and jejunum [9]. Thompson et al. [10] reported that bottle feeding resulted in crypt hyperplasia, with increased crypt length and mitotic count per crypt in duodenum biopsies. It is possible that postnatal diet-associated changes in the GI mucosa will influence the level of mucosal inflammation, autoimmunity, and protection against potential allergy disorders in childhood and adulthood [11–14]. Moreover, breastfed infants have different microbiome composition in comparison to formula fed infants [15–20]. However, the precise mechanism of how the microbial changes in the neonate impact the GI tract and immune system development is not known. The epithelium of GI tract is the first line of defense and plays a major role in maintaining gut membrane integrity. Reports on formula fed rats, rabbits, and neonates showed increased permeability in the GI tract in comparison to breast-fed group [21–24]. Furthermore, it is suggested that components of neonatal diet dictate the microbiome composition and diversity, which could play a role in GI tract membrane protein expression and inflammation status and metabolic signaling. For example, in a monkey model of infant formula feeding at 4 weeks of age, significant up-regulation of IL-1β, IL-1Rα, IL-8, IL-4, GCSF, MIF, CCL3, CCL5, and TNF-α concentrations were observed in serum [25]. It is difficult to determine the inflammatory status of GI tract in infants fed formula diet due to challenges associated with sample collection. Thus, given the widespread use of infant formulas and the effects of these formulas on development and function of the GI tract, it is imperative to develop a better understanding of the implication for the short- and long-term health consequences in children.

Polyamines are known to be involved in functions such as anti-inflammatory role and intestinal epithelial barrier function [26–28]. Interestingly, in breast milk, the concentration of polyamines are 10 times greater than those in formula milk [29, 30]. Polyamines appear to change the gut microbiota composition and influence the gut immune system. In neonatal BALBc mice higher levels of *Bifidobacterium* group, and *Lactobacillus Enterococcus* group were observed with infant formula supplemented with polyamines in comparison to formula alone (3, 4) and, polyamines supplementation in formula influences lymphocyte populations and immune system related gene expression (2). Polyamines have also been shown to have anti-inflammatory effects. For example, in mice oral administration of spermine decreased levels of pro-inflammatory mediator’s nitrate, nitrite, IFN-γ and an increase in anti-inflammatory cytokine IL-10 were observed in serum [28]. Spermidine increased the expression of T cell protein-tyrosine phosphatase (TCPTP), in intestinal epithelial cells and attenuates inflammatory responses [27]. These reports suggest that polyamines play a role in anti-inflammatory process at systemic level in addition to the gastrointestinal tract and are also involved in influencing the gut microbial changes.

To determine the impact of formula diet on GI tract, a neonatal piglet model was employed, because the anatomy and physiology of the pig digestive tract is similar to the human [31–33]. Moughan et al. studies on bovine milk, hydrolyzed bovine milk and soybean formula indicated that pepsin, intestinal trypsin, chymotrypsin were not influenced by protein source and ileal and fecal absorption of nitrogen was similar for both formulas, which supports the use of 3-week-old piglets as a substitute model for 3-month-old human infants [32–34]. The large volumes of tissue harvested from piglets permit the use of different analytical platforms that can be applied, in parallel, to multiple samples from an individual animal, facilitating the adoption of a comprehensive systems approach. The pig is recognized, therefore, as a valuable intermediate between mechanistic research involving laboratory mice and clinical trials in humans [35, 36]. We tested the hypotheses that the formula feeding would alter the gut morphology, gut immune system development, gap junction protein expression and the intestinal microbiota composition using neonatal piglet model.

## Methods

### Animal experiments

Pig experiments were performed as previously described and all animals were housed in the animal facilities of the Arkansas Children’s Hospital Research Institute. Animal maintenance and experimental treatments were conducted in accordance with the ethical guidelines for animal research established and approved by the institutional Animal Care and Use Committee at University of Arkansas for Medical Sciences. All the sow were multiparous and the litter sizes were 8–11. Briefly, White Dutch Landrace Duroc sows were fed a soy-free diet and were artificially inseminated in a commercial farm setting. Piglets were not from the same mother; however, all the mothers and piglets were housed in the same open area, but in separate pens in the same farm. All piglets received 1 ml of intramuscular injection of iron at 2 days old (Iron Hydrogenated Dextran, 100 mg/ml). Piglets were allowed to suckle for 48 h at the farm before being randomly distributed between three groups of approximately equal mean weight. Male and female breastfed piglets (*n* = 6/group/gender) (sow) were placed with sows at the farm for the duration of the experiment in a standard farrowing crate and allowed to breast-feed *ad libitum*. Male and female piglets (*n* = 6/group/gender) that received formula diet were brought to Arkansas Children’s Nutrition Center (ACNC) at 48 h and fed cow’s milk-based formula (milk) (Similac Advance powder; Ross Products, Abbott Laboratories, Columbus, OH) or soy-based formula (soy) (Enfamil Prosobee Lipil powder; Mead Johnson Nutritionals, Evansville, IN). Formula-fed piglets were trained to drink from small bowls on a fixed schedule as described previously to provide 1.047 MJ/kg/day until sacrifice on postnatal day (PND) 21 [37]. Piglets were individually housed, and which allowed us to monitor the food intake and also allowed us to keep the piglets separated in the case of diarrhea. Piglets could see and hear each other but no direct contact and were housed at elevated room temp. They were fed on a strict schedule and all food is consumed within a few minutes: first week was every 2 h, second week was every 4 h, and 3^rd^ week was every 6 h. Each meal is based on a daily desired caloric intake (based on body weight) divided by the number of feeds. The formula diets were modified to meet the energy and nutrient recommendations of the National Research Council (NRC) for growing pigs [38], and the formula diet compositions are described in Additional file 1: Table S1. The sow diet composition has been published previously [39–41] and the comparison of sow diet to formulas is shown in the Additional file 1: Table S1. The formula fed piglets had diarrhea for the first 3–5 days, based on veterinarian recommendation and IACUC requirement, formula diets were prepared in Pedialyte to help with electrolytes loss. Sow fed and formula fed pigs were killed by exsanguination after anesthetization with isoflurane at 0800–1000 h, 6–8 h after the final feeding period. Tissue samples were either snap-frozen in liquid nitrogen or fixed in formalin for histomorphometric evaluation. Frozen tissues were stored at −70 °C until use.

### Histology of small intestine

Formalin-fixed tissues were cut, paraffin-embedded and processed for staining with hematoxylin and eosin (H and E). Prior to histopathology evaluation by a board-certified pathologist slides were coded to blind the reader. All measurements were made using Aperio eslideshare viewer. In order to randomize selection of ileum and jejunum villi and crypts, the low-magnification thumbnail locator was used to select a spot approximately ¼ the way along the section from left to right. The spot was magnified to 5×, and the tallest two villi were measured from the base of the villus to the surface of the epithelium using the pen tool which allows the investigator to follow the curve of the villus. In order to measure, villi had to be intact and entirely within the plane of section. Measurements were repeated at ½ the length of the section, ¾ the length of the section, and near the end of the section. Crypts immediately adjacent or as near as possible to measured villi were selected and crypt depth was measured using the pen tool. Crypts had to be intact and entirely within the plane of section. PP were outlined using the pen tool to generate total area (size). All germinal centers within the follicle area were counted for number of follicles. Within each measured area, the largest three lymphoid follicles were selected and measured. The two largest diameters of each follicle were measured using the ruler tool and averaged. Ileal patch areas were also measured for the overall size, germinal centers and lymphoid follicles. We have measured the perimeter of the mucosa from three different areas from H & E stained ileum sections for each animal with a constant length (400 μm) of ileum with Image J software (ImageJ 1.50b, NIH, USA).. Ratio was calculated by dividing the surface area to the length and reported here as perimeter. To understand the gut mucosa development we measured vacuolization in both jejunum and ileum tissues.

### Membrane protein expression and calcium

Frozen ileum tissue (ca. 100 mg) was homogenized with cell lysis buffer (500 μl) (Cat EPX-99999-000 eBioscience, San Diego, USA) with 0.1 % proteinase inhibitor cocktail (Sigma, St. Louis, MO) and 1 % NP40 in ceramic bead tubes using Fast Prep-24™ 5G machine (M.P. Biomedical LLC, California, USA) at speed 6.0 msec for 30 s (twice), and then centrifuged at 12,000 g for 15 min at 4 °C. Supernatants were collected and 100 μg of protein was used for Western blot analyses. Membranes were probed with 1:1000 dilution of rabbit anti-VE-cadherin, rabbit anti-occludin, and rabbit anti-claudin 3 (Abcam, Cambridge, MA) primary antibodies and subsequently incubated with 1:10,000 dilution of goat-anti-rabbit HRP (BioRad Laboratories Inc., California). Detection was performed using a chemilumiescence system (super signal west chemiluminescent substrate, Thermo Scientific). Immuno-quantitation was performed by densitometric scanning of the blot and normalized against the signal from β-actin (Sigma Aldrich, St Louis, MO) using Image Quant software (Image Quant TL 8.1 Version). We measured calcium levels in urine from breast-fed and formula fed piglets using colorimetric assay from Bio Vision (Catalog#K380-250) as per manufacturer’s instructions.

### Chemokine cytokine analyses

#### Transcript abundance

RNA was extracted from ileum and PP using Trizol reagent. Ileum represents the full thickness along with mucosa. Peyer’s patches were collected from Jejunum. Crude RNA was purified using the Qiagen RNeasy Mini Kit (Qiagen, Valencia, CA) according to the manufacturer’s protocol. One microgram was reverse-transcribed using iSCRIPT cDNA synthesis kit (Bio-Rad, Hercules, CA) following the manufacturer’s instructions. cDNA was diluted to 1:10 and 2 μL was used for quantitative real-time PCR, using SYBR Green master mix (Life Technologies, Grand Island, NY) on a ABI 7500 instrument (Life Technologies, Grand Island, NY). We evaluated four categories of cytokines, interferons (IFNs), interleukins (IL-x), tumor necrosis factors (TNFs), and regulatory cytokines (IL-10 and TGF-β) and four categories of chemokines (CC, CXC, C, and CX3C). Among the different classes of chemokines and cytokines, we measured 56 different chemokines and cytokines that indicate pro-inflammatory, anti-inflammatory, regulatory, and growth aspects of small intestine and GALT tissues. The Ct values were used for each gene and were normalized to the housekeeping gene β-actin to determine the relative expression of chemokines and cytokines. Fold change was determined in formula groups in comparison to the sow fed group. All the primers were designed using primer express software and the accession number and primer sequences for genes quantified by real-time quantitative PCR are given in Additional file 1: Table S2.

#### ELISA

Expression of IL-8, IL-6, and IL-10 were measured in ileum samples of pigs using porcine ELISA kit (EMD Millipore Corporation, MA) as per the manufacturer instructions. Frozen ileum tissue (100 mg) was homogenized with cell lysis buffer (500 μl) (Cat EPX-99999-000 eBioscience, San Diego, USA) in ceramic bead tubes using Fast Prep-24™ 5G machine (M.P. Biomedical LLC, California, USA) at speed 6.0 msec for 30 s, and then centrifuged at 12,000 g for 15 min at 4 °C. Clear tissue supernatant were used to measure the protein expression. Tissue cytokines data was normalized with total protein estimated by BCA method (Thermo-scientific IL, USA) and data are presented as pg/g protein.

### UHPLC-HRAM analysis of polyamine concentrations in pig ileum

Optima grade acetonitrile, methanol, formic acid, and water were purchased from Fisher Scientific (Pittsburgh, PA). Spermidine-trihydrochloride, spermine, putrescine, and lorazepam standard compounds were obtained from Sigma-Aldrich (St. Louis, MO). Ileum tissue (25 mg) was homogenized in 1 mL 50 % aqueous methanol using a PowerGen 1000 homogenizer (Fisher Scientific, Pittsburgh, PA). Compounds were extracted in 2 mL acetonitrile, dried under a nitrogen stream, and then reconstituted in 300 μL of 5 % aqueous methanol spiked with internal standard. Chromatographic separation was performed on an UltiMate 3000 UHPLC system (Thermo Scientific, Sunnyvale, CA) fitted with a Hypersil GOLD C18 reversed phase column (50 × 2.1 mm, 1.9 μ) kept at 30 °C. Mobile phases consisted of 0.1 % formic acid in water (A) and 0.1 % formic acid in acetonitrile (B). Elution gradient, flow rate, and injection volume were as previously described [42]. Polyamine identification was carried out on a Q Exactive high-resolution accurate mass (HRAM) spectrometer (Thermo Scientific, Sunnyvale, CA) with data acquisition and analysis performed using Xcaliber (version 2.2) software. Data was acquired by positive electrospray ionization (ESI+) Full-MS scan mode. Nitrogen as sheath, auxiliary, and sweep gas was set at 50, 13, and 3 units, respectively. Other conditions included: resolution, 70,000 FWHM; AGC target, 3e6 ions; maximum injection time, 200 ms; scan range, 50–750 m/z; spray voltage, 3.50 kV; and capillary temperature, 320 °C. Mass accuracy within < 5 ppm plus a retention time within 30 s of a calibrator was used for peak confirmation. Peak area measurements were used to quantify polyamine amounts using standard curves based on spermidine, spermine, and putrescine and normalized using Lorazepam as an internal standard.

### 16sRNA amplicon sequencing

Ileum contents were subjected to DNA isolation and sequencing as described by Kozich et al. [43]. DNA was extracted using Qiagen DNA isolation kit, amplicons generated by PCR of variable region 4 (V4) of bacterial 16S rRNA genes and multiplex sequencing was carried out with an illumina platform. Clustering of V4 rRNA reads at 97 % nucleotide sequence was performed using QIIME software.

### Statistical analyses

Group comparisons were assessed by Kruskal Wallis tests and interaction among groups and gender were assessed by non-parametric 2-factor ANOVA. 1-factor ANOVA was used to determine the significance for mucosal surface area data. Negative binomial regression analyses was used for vacuolization data. Two tailed t-test was used to calculate significance for ELISA, polyamine, E-cadherin expression and microbial spp data between sow and milk groups. Data were presented as mean ± SEM and statistical significance was determined at *P* < 0.05.

## Results

### Body weights

Body weights at sacrifice and growth rates of piglets from this experiment at day 21 did not differ significantly between the three neonatal diet groups and no gender differences were observed (Additional file 1: Table S3).

### Gastrointestinal tract development

#### Small intestine length and mucosa

**Fig. 1:**
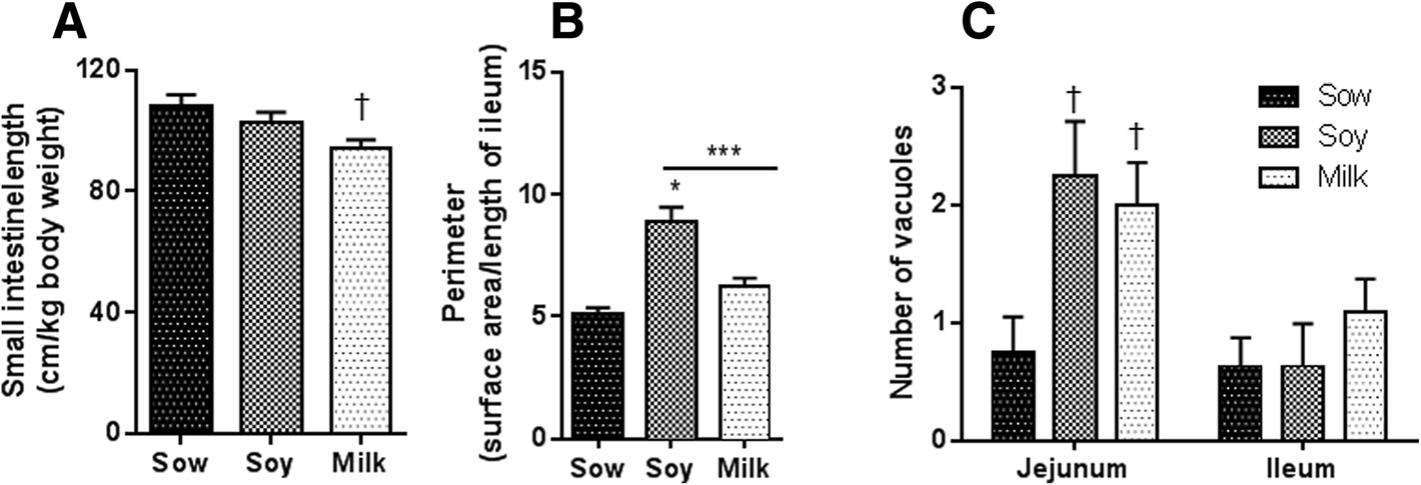
Data are a pool of 6 males and 6 female piglets for each diet group and mean ± SEM from 12 animals are plotted for each diet group. **a** Small intestine length from piglets fed three different infant diets (sow milk, soy or cow’s milk formula) is reported as cm/kg body weight. Non parametric 2-factor ANOVA analyses of data indicated a group difference but no interaction or gender differences (*p* < 0.01 diet, *p* < 0.58 sex, *p* < 0.51 interaction). Milk fed animals showed significant differences in comparison to sow-fed (KW ^†^ *p* < 0.01). **b** Perimeter of the mucosa was calculated as explained in the methods section. One-factor ANOVA analyses of data showed significant differences between sow and soy (*P* < 0.0001) and sow and milk (*P* < 0.1, at tenth percentile*)*. **c** Histomorphometric analyses of jejunum and ileum vacuoles from piglets fed three different infant diets (sow milk, soy or cow’s milk formula). Negative binomial regression analyses indicated statistical significance among different diet groups (*p* < 0.01) in jejunum

Non-parametric 2-factor ANOVA analyses indicated a group difference, but no interaction or gender differences and hence the data were pooled from both male and female piglets (Fig. 1). The length of the small intestine was normalized to the body weight of the animals. Sow-fed piglets had significantly longer small intestine in comparison milk-fed piglets and a trend in soy. The decrease in small intestine length in formula groups corresponds to 6 % in soy- and 14 % in milk-fed piglets; the latter change achieved statistical significance (Fig. 1a). We have also measured the surface area of the mucosa and significantly increased surface area was observed in soy piglets in comparison to sow fed piglets and only a trend in increased surface area with milk group in comparison to sow (Fig. 1b). Based on previous reports, abundance of vacuoles in enterocytes may be indicative of inhibition in cell turnover and maturations [44], and the presence of vacuoles can track changes in mucosa development [45]. Thus, the number of vacuoles in ileum and jejunum were determined (Fig. 1c). No statistically significant differences were observed in ileum among the different diet groups (Fig. 1c). However, in jejunum with both soy and milk formula diets, a significantly increased number of vacuoles was observed suggesting an impact on mucosa development.

#### Histomorphological analyses and Peyer’s patch

**Fig. 2:**
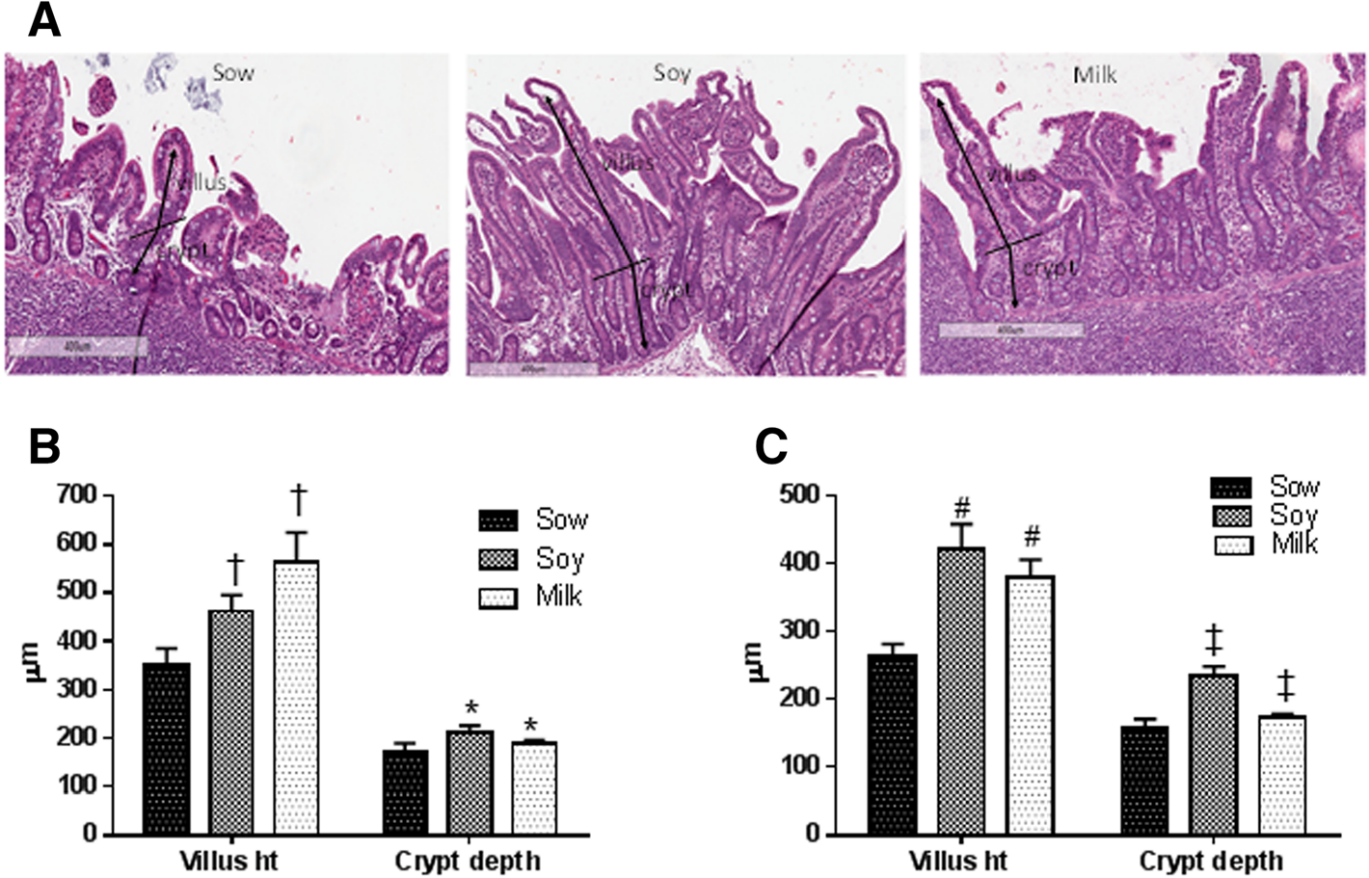
Histomorphometric analyses of jejunum and ileum from piglets fed three different infant diets (sow milk, soy or cow’s milk formula). Data are a pool of 6 males and 6 female piglets for each diet group and mean ± SEM from 12 animals are plotted for each diet group. Villus height and crypt depth are indicated in micrometers on *Y-axis*. Non parametric 2-factor ANOVA analyses of data indicated a group difference but no interaction or gender differences. **a** Representative pictures of ileum villus height and crypt depth of sow, soy and milk fed piglets. Villus height and crypt depth are indicated with arrows. **b** Jejunum statistical analyses for villus height (*p* < 0.01 diet, *p* < 0.26 gender, and *p* < 0.71 interaction) and crypt depth (*p < 0.05 diet, *p* < 0.67 gender, and *p* < 0.14 interaction) indicates increased villus height and crypt depth in formula groups in comparison to sow fed (KW ^†^ *p* < 0.01 villus height, *p < 0.05 crypt depth). **c** Ileum statistical analyses for villus height (*p* < 0.0008 diet, *p* < 0.33 gender, and *p* < 0.43 interaction), crypt depth (*p* < 0.0005 diet, *p* < 0.12 gender, and *p* < 0.95 interaction) showed increased villus height and crypt depth in formula groups in comparison to sow fed (KW #*p* < 0.001 villus height, ^‡^ *p* < 0.005 crypt depth)

In order to understand the impact of formula on small intestine development, formalin-fixed jejunum, ileum, and PP tissues were processed for histomorphological analyses. Non parametric 2-factor ANOVA analyses of data indicated a group difference, but no interaction or gender differences with jejunum, ileum or PP; hence, the data were pooled from both male and female piglets. Both soy- and milk-fed piglets showed significantly increased villus height and crypt depth in jejunum in comparison to sow-fed piglets (Fig. 2a, b). It has to be noted that there was 32 and 62 % increase in villus height with soy or milk formula diets, respectively, in comparison to sow-fed piglets. Similarly, there was a 23 and 10 % increase in crypt depth in soy- and milk-fed piglets, respectively, compared to sow-fed piglets. Ileum histomorphological analyses also showed increased villus height and crypt depths in soy- and milk-fed piglets in comparison to sow-fed piglets (Fig. 2a, c). The percent increase in villus height was 59 and 44 % in soy- and milk-fed piglets, respectively. Similarly crypt depth increases were 48 and 10 % in soy- and milk-fed piglets, respectively.

**Fig. 3:**
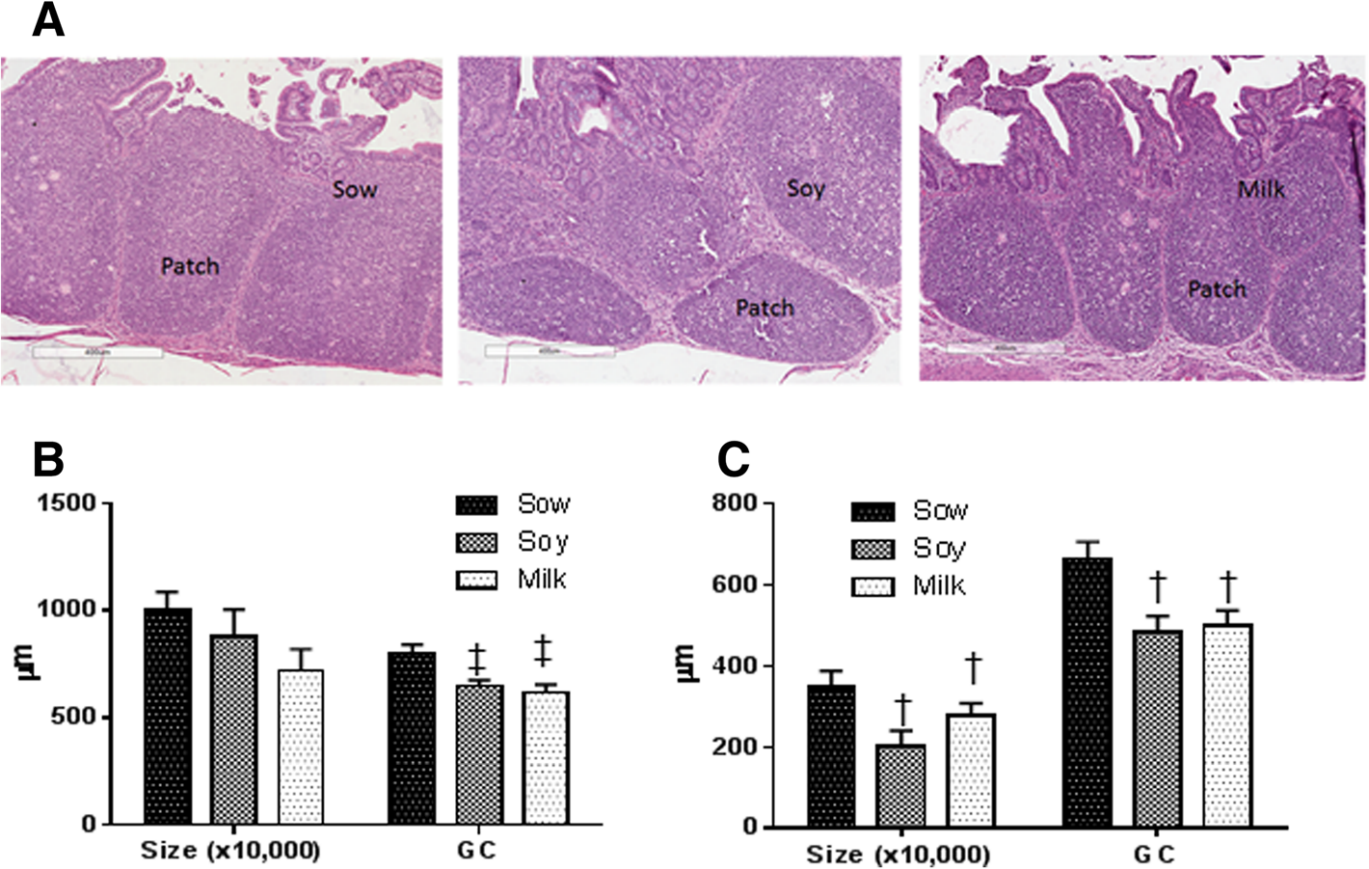
Histomorphometric analyses of ileum and jejunum lymphoid follicle. Data are a pool of 6 males and 6 female piglets for each diet group and mean ± SEM from 12 animals are plotted for each diet group. Non parametric 2-factor ANOVA analyses of data indicated a group difference but no interaction or gender differences. The entire area of the lymphoid follicle from ileum and jejunum area were measured which is indicated as size. The follicle size is 10,000 times to the *Y-axis* (μm). **a** Representative pictures of ileum lymphoid follicle of sow, soy and milk fed piglets. Sow fed piglets show bigger follicle than soy or milk fed piglets. **b** No significant differences were observed in size of the follicle within the Ileum with different diets (*p* < 0.14 diet, *p* < 0.54 gender and *p* < 0.18 interaction). However significant differences were observed in the diameter of germinal centers (GC) measured (*p* < 0.0005 diet, *p* < 0.06 gender and *p* < 0.3751 interaction) in the ileum. **c** Similarly to ileum the entire area of the lymphoid follicle of jejunal PP was measured which is indicated as size and significant differences were observed in size of the follicle in jejunal PP with different infant diets (*p* < 0.009 diet, *p* < 0.70 gender and *p* < 0.37 interaction). In addition, significant differences were observed in the diameter of germinal centers (GC) measured (*p* < 0.005 diet, *p* < 0.7 gender and *p* < 0.54 interaction). KW p values are indicated on the graphs of ileum and PP in soy and milk formula groups with respect to sow fed (^†^ *p* < 0.01, ^‡^ *p* < 0.005) on the graphs

To further determine the impact of formula on lymphoid follicle development in the small intestine, the area of lymphoid follicle (size) and follicle germinal center diameter were measured in ileum and jejunum lymphoid patch (Fig. 3). Both in ileum and PP in comparison to sow-fed piglets, soy- or milk-fed piglets showed decreased follicle size and germinal centers with no difference in number of follicles observed. There was no statistical significance for ileal follicle size among the groups, but there was a 12 and 28 % decrease in follicle size with soy or milk diet in comparison to sow-fed diet (Fig. 3b). However, a statistically significant decrease in germinal center diameter was observed among different groups with 20 % decrease (*P <* 0.005) in soy and 24 % decrease (*P <* 0.005) in milk diet piglets in comparison to sow-fed piglets (Fig. 3b). Similarly, the jejunal PP lymphoid follicle size was significantly lower in soy- and milk-fed piglets in comparison to sow diet (Fig. 3c). We observed a 42 and 20 % decrease in soy- and milk-fed piglets, respectively (*P <* 0.01), compared to sow-fed piglets. Even germinal centers sizes were decreased, 27 % in soy-fed (*P <* 0.005) and 25 % in milk-fed piglets (*P <* 0.005), respectively, compared to sow-fed (Fig. 3c). We did not observe any significant differences between soy and milk formula groups with gut morphology; thus, most of our parameters were measured only in sow and milk formula fed groups from here on.

### Membrane protein expression and calcium levels

**Fig. 4:**
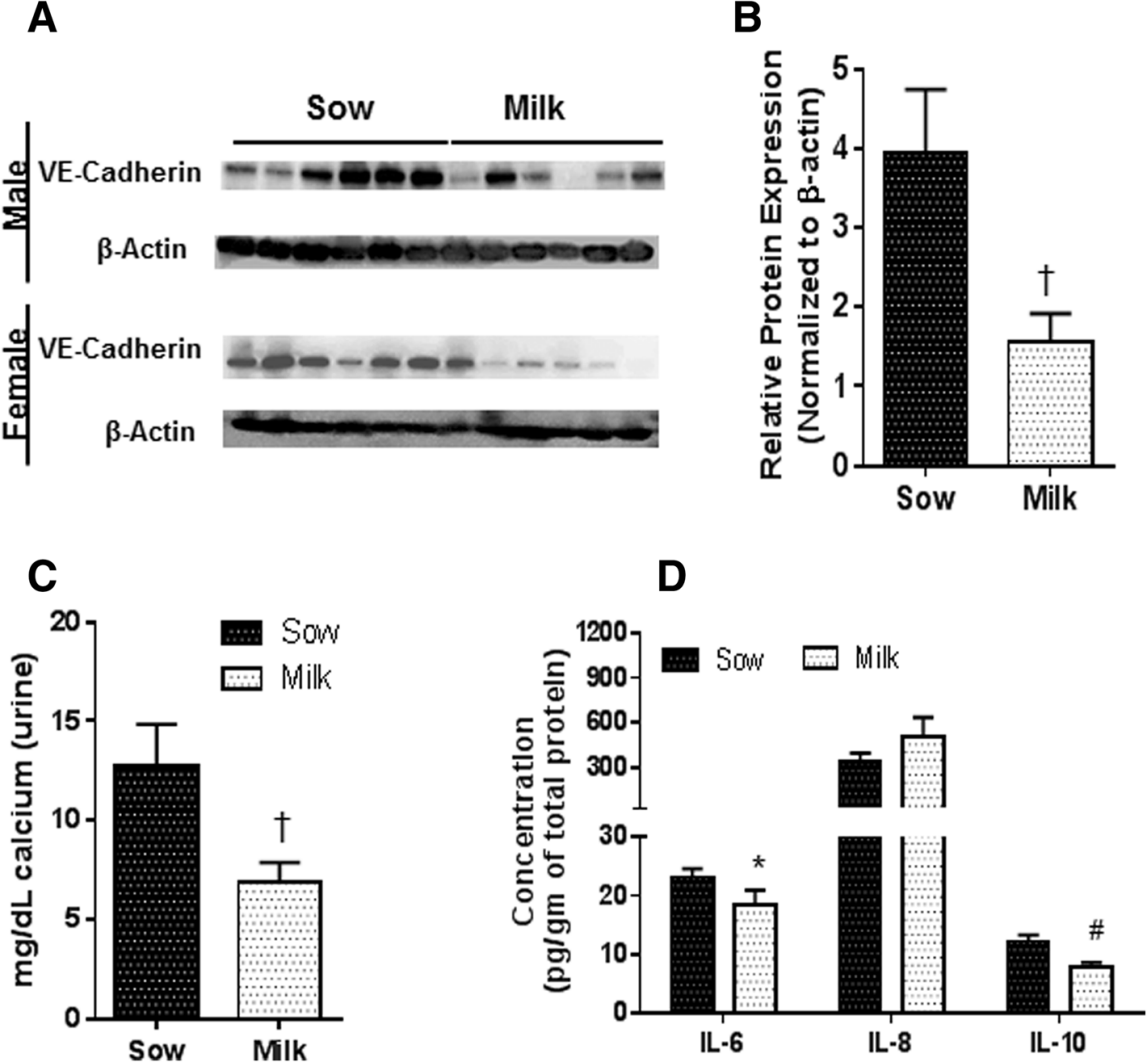
Data are a pool of 12 piglets (6/gender) for each diet group for calcium and protein data. Data are presented as mean ± SEM and are analyzed by 2-tailed t –test. **a** Western blot image of E-cadherin. **b** Relative expression of VE-Cadherin showed significantly lower levels in milk group in comparison to sow (^†^ *p* < 0.01) **c** Calcium **d** Ileum protein expression of milk group showed significant difference with IL-10 (#*P* < 0.001), IL-8 (*P* < 0.1, at tenth percentile) and IL-6 (*P < 0.05,) in comparison to sow

To determine if milk formula diet alters the gut membrane protein expression we measured VE-cadherin, occludin and claudin-3. We observed significantly higher amount of VE-cadherin in sow-fed piglets in comparison to milk formula-fed piglets (Fig. 4a and b), no significant differences were observed with occludin or claudin-3 (data not shown). Expression of VE-cadherin is tightly regulated to calcium levels and to determine this, urine calcium levels were measured. Significantly higher amount of calcium was observed in sow fed group in comparison to milk formula group (Fig. 4c).

### Differential expression of chemokines and cytokines

#### Transcript abundance

In ileum with soy or milk diet, significant differences with 16 chemokines and cytokines were observed when compared to sow diet (Table 1 and Additional file 1: Figure S1). Transcripts for pro-inflammatory molecules AMCFII, IL-8, IL-15, VEGFA, LIF, FASL, CCL4, and CCL25 were significantly up-regulated in formula fed piglets in comparison to sow-fed piglets. Furthermore, a 1.5-fold decreased expression of anti-inflammatory molecule IL-10 and 3 to 20-fold decreased expression of IFNα-4, IL-6, IL-9, IL-27, CSF3, LOC100152038 and LOC100736831 (Table 1 and Additional file 1: Figure S1) were observed in soy- and milk-fed piglets. The largest decrease was observed with IL-9 in soy and milk-fed piglets.

**Table 1:** Chemokines and cytokines mRNA expression of piglets. Data are from 6 male and 6 female piglets for each diet group. Data are presented as fold change ± SEM and fold changes are calculated with respective to sow group (1). Nonparametric 2-factor ANOVA analyses was carried out to determine the statistical significance

Transcript	Soy	Milk	Sow vs Soy (*P*)	Sow vs Milk (*P*)	Soy vs Milk (*P*)
AMCFII	4.6 ± 1.0	2.1 ± 0.3	0.0005	0.001	0.005
IL-8	6.2 ± 3.1	4.5 ± 2.3	0.001	0.05	NS
IL-15	2.1 ± 0.4	1.6 ± 0.4	0.005	0.05	NS
VEGFA	2.1 ± 0.5	1.6 ± 0.4	0.05	0.05	NS
LIF	2.9 ± 0.8	2.1 ± 0.5	0.005	0.005	NS
FASL	2.6 ± 0.8	1.6 ± 0.3	0.01	0.05	NS
CXCL11	1.1 ± 0.6	−4.2 ± 0.1	NS	0.05	NS
CCL4	2.6 ± 1.0	2.2 ± 0.6	0.050	0.05	NS
CCL25	2.5 ± 1.4	2.0 ± 1.0	0.050	0.05	NS
IL-6	−3.1 ± 0.15	−2.9 ± 0.16	0.005	0.005	NS
IL-9	−24 ± 0.03	−30 ± 0.02	0.0005	0.005	NS
IL-10	−1.5 ± 0.13	−1.4 ± 0.12	0.05	0.05	NS
IL-27	−4.5 ± 0.12	−10.6 ± 0.04	0.001	0.0001	NS
IFNA4	−4.0 ± 0.12	−4.7 ± 0.09	0.001	0.0005	NS
CSF3	−8.1 ± 0.06	−11.7 ± 0.05	0.0005	0.0005	NS
LOC100152038	−5.8 ± 0.12	−13.9 ± 0.04	0.005	0.0005	NS
LOC100736831	−5.8 ± 0.06	−4.9 ± 0.08	0.0005	0.0005	NS

#### Cytokine and chemokine protein expression

To validate our transcript data we have carried out protein ELISA to measure IL-6, IL-8 and IL-10 from ileum tissue for sow and milk groups as we did not observe any significant difference between soy and milk. We observed significant down regulation of anti-inflammatory molecule IL-10 and IL-6 in both ileum and serum of milk fed piglets in comparison to sow fed piglets (Fig. 4d). There was a trend in increased expression of pro-inflammatory molecule IL-8 in ileum in milk group in comparison to sow group (*P* < 0.07)).

### Polyamines

**Fig. 5:**
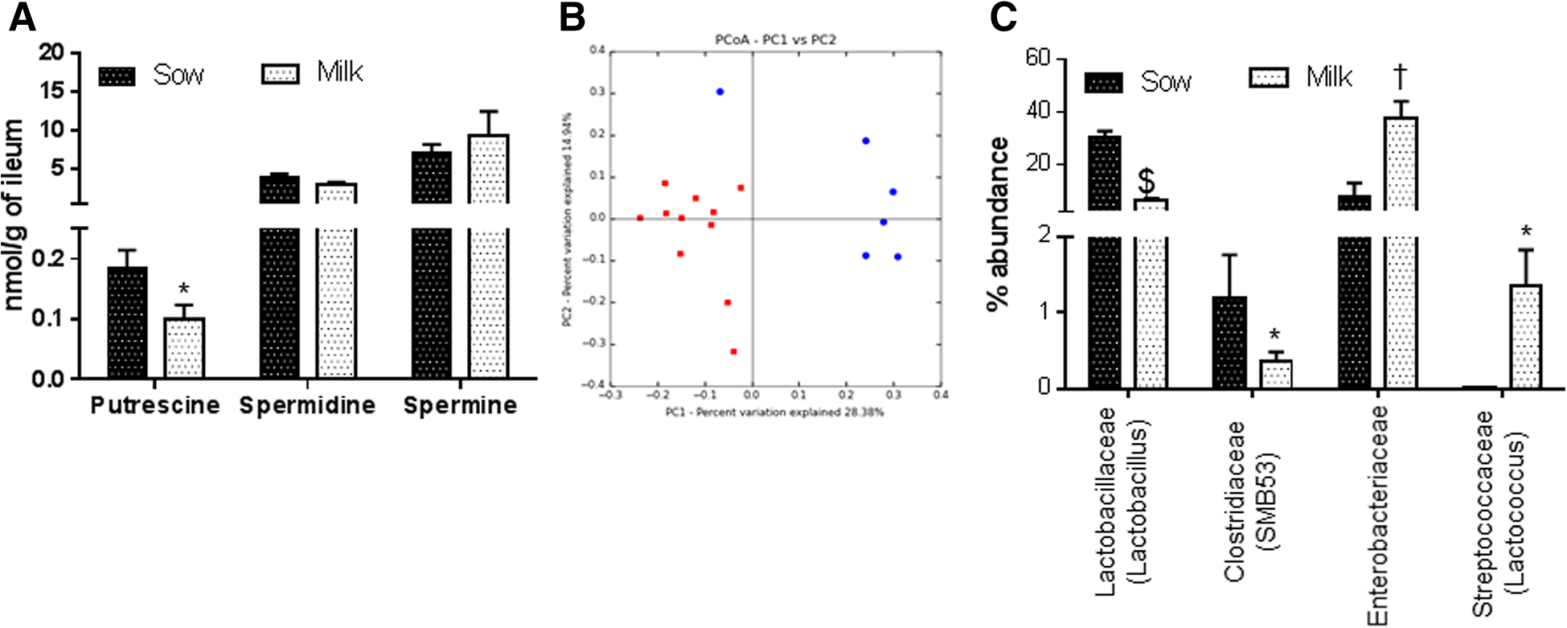
Data are a pool of 12 piglets (6/gender) for each diet group for polyamine data. Microbiome data for milk group N is 11 (5 males and 6 females) and for sow group N is 6 (3 males and 3 females). Data are presented as mean ± SEM and are analyzed by 2-tailed t –test. **a** Ileum tissue polyamine measurements showed significantly higher Putrescine (*p < 0.05), spermidine (*p < 0.07, at tenth percentile) and no significant differences with spermine in sow fed piglets in comparison to milk formula fed piglets. **b** PCoA plot of ileum microbiome **c** Ileum contents microbiome analyses showed significant differences between sow and milk fed piglets with Lactobacillaceae spp ($*P* < 0.000001), Clostridiaceae spp (*p < 0.05), Enterobacteriaceae spp (^†^ *p* < 0.01), and Streptococcaceae spp (*p < 0.05)

Previously it has been shown that 10 times higher levels of polyamines are present in breast milk than in formulas [29, 30] and E-cadherin expression is regulated with polyamines [26]. To determine the levels of polyamines in ileum tissue of sow and milk fed piglets (Putrescine, spermidine, and spermine) samples were processed for targeted analyses. We observed significantly higher levels of putrescine in ileum of sow fed piglets in comparison to milk group (Fig. 5a). There was no statistical significant difference observed with spermidine or spermine.

### Ileum microbiome

Ileum contents were processed for evaluating microbial richness and diversity as explained in methods section. The OTU table was used for generating alpha and beta-diversity. Taxa summary tables and principal component analyses (PCoA) plots of beta-diversity were also generated using QIIME. No significant differences were observed with alpha diversity as measured by PD whole tree, observed OTUs and chao1 analyses between sow and milk groups. However, PCoA of beta-diversity values indicated clear separation of the diet groups for ileum microbiome (Fig. 5b, *p* < 0.005 Bonferroni corrected). Taxa summary tables of beta-diversity at the family level indicated Lactobacillaceae spp being the most abundant and Clostridia spp the next abundant in sow fed piglets. In comparison to formula fed piglets, sow fed piglets showed 5-fold higher Lactobacillus spp at genus level within Lactobacillaceae family. In Clostridiaceae family, SMB53 genus was 3-fold higher in sow-fed piglets in comparison to milk formula fed piglets (Fig. 5b). Interestingly in milk formula fed piglets at the family level Enterobacteriaceae spp was the most abundant with 5-fold higher amounts in comparison to sow group. Furthermore, in Streptococcaceae family, Lactococcus spp was the most predominant genus in milk group (Fig. 5c).

## Discussion

To understand the role of early diet on the GI tract, we have leveraged a piglet model to comprehensively characterize small intestine and GALT development, membrane protein expression, inflammation status and microbial changes in piglets fed infant formulas (soy-based and milk-based) and compared the effects to breastfed piglets. Previous reports on piglets that were fed formula for 21 days starting at postnatal day 7 showed increased mucosal wall thickness and greater density (g/cm) in the ileum and jejunum [9]. The current study addressed the effects of breastfeeding for the immediate post-delivery 48 h followed by a switch to formula for the next 20 days, because this situation is relevant the sub-group of human newborns in whom postpartum breastfeeding is the similar length.

Our data demonstrated that the small intestine is longer in suckled piglets in comparison to formula-fed piglets. Formula-fed piglets have significantly longer villi and deeper crypts in comparison to suckled piglets on day 21 in both ileum and jejunum, which supports the limited observations reported for human infants [10]. The authors obtained duodenum biopsies by endoscopic techniques from healthy infants (age 2–6 months) who were exclusively breastfed or formula-fed. Formula-fed infants showed increased crypt depth by 30 % with no difference in villus height in comparison to breastfed infants [10]. Jensen et al. reported significantly increased mucosal weights and crypt depth in two day old formula-fed piglets in comparison to two day old piglets fed porcine colostrum [46]. Thyman et al. showed reduced lactose digestive capacity in piglets fed formula at 24 h, compared to sow-fed animals, and suggested that formula feeding exerts detrimental effects on intestinal function [47]. De Vos et al. reported increased crypt depth, increased small intestine length and decreased spleen size with 7 days formula feeding starting from day 3 of life in piglets in comparison to sow-fed [48]. Most recently, Sugiharto et al. conducted experiment with three week old sow fed piglets and fed formula or bovine colostrum or left on sow for 8 days [49]. The formula fed piglets showed higher frequency of diarrhea, lower lactic acid bacteria to E.coli ratio and lower mucosal IgG than the sow fed or bovine colostrum group. Taken together with the length of small intestine, villus height, and crypt depth of ileum and jejunum and surface area of the mucosa, our data support the idea that infant formula diet alters small intestine morphology, which fits with previous reports [47–49]. In addition, significantly more vacuoles were observed in jejunum of formula-fed piglets and these results are in agreement with the previous report, where piglets fed formula diet only for 7 days showed higher abundance of vacuoles in mid-jejunum [45]. It is confirmed from these reports, including ours, that formula feeding even for a short period possibly results in altered small intestine and possibly delays GI tract development. Since growth, as measured by body weight, did not differ between breastfed and formula-fed piglets, the long-term consequences of GI morphology alteration by formula feeding are not yet clear. Factors beyond body weight gain, such as immune system development and intestinal permeability, may be important.

The current study examined GALT development and the role of early diet (formula vs breastfeeding) on the immune system. Significantly smaller sized follicles with smaller germinal centers, both in ileum and PP, were observed with soy or milk diet in comparison to sow-fed piglets. The data indicated less well developed small intestine lymphoid tissue in which further confirms our previous report of neonatal piglets fed with casein-based diet showed less numerous PP (13). Furthermore, we did not observe any significant differences between soy and milk formula fed piglets with gut morphology, suggesting that soy or milk formula alter GI tract similarly. Previous reports have indicated that breastfed infants are at less risk to upper respiratory tract infections, ear infections, and gastrointestinal illnesses in the first year of life [9]. Thus, our results in the piglet model are consistent with clinical data in children indicating that formula feeding impacts the gut immune system development, and raises the possibility that formula-fed infants could have lower and delayed immune responses to infections early in life when compared to breastfed infants.

Previously it has been reported that formula feeding could impact membrane permeability and possibly rearrange the membrane proteins [21–24]. To determine the tight junction protein expression we measured membrane protein expression of E-cadherin, Occludin and claudin-3. We observed significantly higher expression of E-cadherin but no changes with claudin-3 or Occludin. E-cadherin expression is tightly dependent on calcium, thus levels of calcium were measured and determined that significantly lower levels of calcium were present in urine in formula group in comparison to sow. It is important to note that in formula twice amount of calcium is present in comparison to sow milk; thus, the lower amount of calcium in urine suggests that additional calcium is possibly being excreted out via the feces in formula-fed animals. Previously, piglets fed formula diet from d7 to d28 did not show any difference in permeability using lactulose/mannitol assay but interestingly tight junction protein ZO-1 involved in membrane permeability showed a 2-fold decrease in expression in comparison to suckling piglets. Even though we have not carried out functional assay for membrane permeability, our results on calcium dependent protein VE-cadherin indicated decrease in expression in milk formula fed group in comparison to suckling piglets and calcium data supported the cadherin expression. Data from our study and others confirms that gut membrane protein expression is altered, which could in theory increase risk for systemic exposure to microbial antigens that can possibly impact the education of immune system especially to oral tolerance and consequences later in life. Previous report has indicated that polyamines regulate E-cadherin through calcium [26], and 10 times higher levels of polyamines are present in breast milk than in formulas [29, 30]. Thus levels of polyamines were measured in ileum tissue samples of sow and milk groups. Significantly lower levels of putrescine were observed in milk group in comparison to sow. These data further support that levels of polyamines possibly regulate the membrane protein E-cadherin expression through calcium and possibly could increase the membrane permeability in the GI tract. Overall further mechanistic studies are needed to determine the extent gut membrane function is comprised in formula fed piglets.

Polyamines have been shown to have anti-inflammatory effects both in vitro and in vivo models, where Spermidine increased the expression of T cell protein-tyrosine phosphatase (TCPTP), in intestinal epithelial cells and attenuates inflammatory responses [27]; and in elderly people administration of *Bifidobacterium lactis* LKM512 in yogurt suppressed intestinal inflammation which correlated with increased fecal putrescine, spermidine and cadaverine levels [50] suggesting that levels of polyamines in the ileum could regulate the inflammatory molecule expression in the tissue. Several reports have indicated that formula diet increases inflammatory molecule expression [49, 51]. For example, in type 1 diabetes rat model, animals were formula-fed only for 7 days from day 7 of life and switched back to mother’s milk until day 21, and inflammatory molecule expression was measured on day 75 [52]. Those rats showed increased pro-inflammatory molecule (TNF-α, IFN-γ, and IL-18) expression in liver but no changes in the GI tract. It is possible that in these rats the changes in the GI tract have happened earlier than day 75. Moreover, piglets in the current study received formula for 19 days in comparison to the rat model where only one week of formula was received and responses were measured at the end of the formula feeding unlike in rat model [52]. Most recently Schwartz et al. with the integrated mRNA analyses of sloughed off epithelial cells from GI and microbiota (fecal samples), showed down-regulation of genes that prime mucosal inflammatory responses such as KLRF1 (killer cell lectin-like receptor subfamily F member), BPL1 (permeability increasing protein like), ALOX5 (arachidonate 5-lipoxygenase), IL-1α (interleukin 1 alpha), and AOC3 (vascular adhesion protein 1) in exclusively breastfed infants but not in exclusively formula-fed infants at 3 months of age, again suggesting that formula diet has impact on GI tract inflammatory responses [51]. In our study, formula feeding resulted in a significant increase in expression of neutrophil chemoattractants AMCFII (alveolar macrophage-derived chemotactic factor II) and IL-8 (mRNA and protein), IL-15, LIF, FASL, CXCL11, CCL4 and CCL5 and significant downregulation of anti-inflammatory molecule IL-10 (mRNA and protein) suggesting that formula diet alters inflammatory and anti-inflammatory responses.

Polyamine-supplemented formula diet in BALB/c mouse model showed increased abundance of Bifidobacteria and Lactobacillus, which fits with our data where the most abundant species observed was Lactobacillaceae at the family level in sow-fed piglets. These data suggest that diet components of breast milk enrich specific microbial populations, which in turn could also regulate the polyamines levels. However, it does not rule out the possibility that other components of breast milk such as growth factors, hormones, milk oligosaccharides and ions that can modulate signaling mechanism in infants could possibly reduce inflammatory reactions, such as TNF superfamily glycoprotein osteoprotegerin, a cytokine receptor which has been shown to reduce TNF-induced T-cell proliferation thereby reducing inflammatory response [53]. Hence, the components present in breast milk in addition to polyamines, would also protect the infant’s gut from inflammatory responses. Further studies are required to determine mechanism (s) involved in GI tract and immune system development in association with neonatal diet.

## Conclusions

In summary, the data from our study raise the possibility that the initial up-regulation of pro-inflammatory molecules, alterations in membrane morphology, protein expression and gut microbial changes observed in piglets that are exclusively formula-fed can have a profound impact on the gut immune system. If recapitulated in humans, these postnatal diet-associated alterations in GI and immune system development could have long-term health consequences.

## Limitations and future studies

A strength of the current study is the use of the piglet model, which is a robust one for studying events relevant to infant GI tract development. It is acknowledged in the current study that piglets fed sow milk were at the farm and the environment is different from piglets fed formulas, which may have impacted GI microbial populations in addition to the diet. Although the piglets are not from the same mother, we think there is very minimal impact of mother’s housing environment and mother’s microbiome contribution in sow fed piglets, as there are all housed in an open area in the same farm in separate pens with same diets. The microbiota that is acquired in early life due to differences in diet or environmental exposure has been proven to be important for mucosal immune response and tolerance; hence, alterations of gut microbiota will directly influence the mucosal inflammation, autoimmunity, and allergy disorders in childhood and adulthood [11–14]. Thus, the role of the microbiota on pro-inflammatory and regulatory functions due to differences in infant diets is the scope of future work. Furthermore, microbiome may take several weeks to fully manifest, thus future studies will be considered in older animals. Additional work to compare the outcomes from the current study with animals fed human breast milk and formula in a laboratory setting will be important.

## Additional file

Additional file 1: Table S1. Diet composition of milk, soy and sow. **Table S2.** Primers or probes were designed using primer express software. **Table S3.** Body weights at day 20. Statistical analyses of data was carried out by mixed effect regression model. Data are presented as mean±SEM. **Figure S1.** Chemokine cytokine data are shown as heat map. We have 12 animals/group and 6/gender. Data are normalized to unit variance. Generated in the R statistical environment. (DOCX 314 kb)
