# Erratum to: Formula diet alters small intestine morphology, microbial abundance and reduces VE-cadherin and IL-10 expression in neonatal porcine model

**DOI:** 10.1186/s12876-016-0469-5

**Published:** 2016-05-26

**Authors:** Laxmi Yeruva, Nicole E. Spencer, Manish K. Saraf, Leah Hennings, Anne K. Bowlin, Mario A. Cleves, Kelly Mercer, Sree V. Chintapalli, Kartik Shankar, Roger G. Rank, Thomas M. Badger, Martin J. J. Ronis

**Affiliations:** Arkansas Children’s Nutrition Center, 15 Children’s Way, Little Rock, AR 72202 USA; Arkansas Children’s Hospital Research Institute, Little Rock, USA; Department of Pediatrics, University of Arkansas for Medical Sciences, Little Rock, USA; Department of Pathology, University of Arkansas for Medical Sciences, Little Rock, USA; Department of Pharmacology & Experimental Therapeutics, Louisiana State University Health Sciences Center, New Orleans, LA USA

## Erratum

Following publication of the original version [1] of the article in BMC Gastroenterology, it was brought to our attention that there was a mistake in the references list.

Please see updated references below:

32. Gomez-Gallego C, Collado MC, Perez G, Ilo T, Jaakkola UM, Bernal MJ et al. Resembling breast milk: influence of polyamine-supplemented formula on neonatal BALB/cOlaHsd mouse microbiota. Br J Nutr 2014;111:1050-8.

33. Gomez-Gallego C, Collado MC, Ilo T, Jaakkola UM, Bernal MJ, Periago MJ et al. Infant formula supplemented with polyamines alters the intestinal microbiota in neonatal BALB/cOlaHsd mice. J Nutr Biochem 2012;23:1508-13.

34. Gomez-Gallego C, Frias R, Perez-Martinez G, Bernal MJ, Periago MJ, Salminen S et al. Polyamine supplementation in infant formula: Influence on lymphocyte populations and immune system-related gene expression in a Balb/cOlaHsd mouse model. Food Research International 2014;59:8-15.

The above references should be cited in the following text as below:

“Polyamines appear to change the gut microbiota composition and influence the gut immune system. In neonatal BALBc mice higher levels of *Bifidobacterium* group, and *Lactobacillus Enterococcus* group were observed with infant formula supplemented with polyamines in comparison to formula alone and, polyamines supplementation in formula influences lymphocyte populations and immune system related gene expression [32-34]”.
