# Supplementary material for: Formula diet alters small intestine morphology, microbial abundance and reduces VE-cadherin and IL-10 expression in neonatal porcine model
Source: BMC Gastroenterol. 2016 Mar 22;16:40. doi: 10.1186/s12876-016-0456-x (PMC4804644; doi:10.1186/s12876-016-0456-x)

Additional file 1

| Table S1: Diet composition of milk, soy and sow. ND = not determined. | | | | |
| --- | --- | --- | --- | --- |
| **Nutrient** | **unit** | **Milk diet/liter** | **Soy diet/liter** | **Sow diet/liter** |
| Protein | g | 69.8 | 68.3 | 56.0 |
| Carbohydrate | g | 116.5 | 131.7 | 48.0 |
| Fat | g | 53.1 | 59.4 | 78.0 |
| ***Essential amino acids*** | | | | |
| Arginine | g | 2.845 | 5.779 | 3.25 |
| Histidine | g | 1.885 | 1.832 | 1.23 |
| Isoleucine | g | 4.251 | 3.289 | 2.41 |
| Leucine | g | 8.876 | 5.702 | 4.59 |
| Lysine | g | 6.979 | 5.650 | 4.19 |
| Methionine | g | 1.850 | 1.729 | 0.77 |
| Methionine + Cystine | g | 3.091 | 3.612 | 1.712 |
| Phenylalanine | g | 3.489 | 3.818 | 1.98 |
| Phenylalanine + Tyrosine | g | 7.143 | 6.527 | 4.241 |
| Threonine | g | 3.981 | 3.599 | 2.01 |
| Trytophan | g | 1.311 | 1.084 | 0.873 |
| Valine | g | 4.859 | 3.754 | 2.86 |
| ***Non-Essential amino acids*** | | | | |
| Cystine | g | 1.241 | 1.883 | 0.828 |
| Tyrosine | g | 3.653 | 2.722 | 2.148 |
| Alanine | g | 3.208 | 3.096 | 1.533 |
| Aspartic Acid | g | 7.471 | 8.308 | 4.353 |
| Glutamic Acid | g | 15.714 | 14.087 | 12.264 |
| Glycine | g | 1.628 | 2.954 | 1.264 |
| Proline | g | 6.534 | 4.425 | 6.042 |
| Serine | g | 4.087 | 3.689 | 2.965 |
| ***Minerals*** | | | | |
| Calcium | mg | 3155.39 | 3615.61 | 1630 |
| Phosphorous | mg | 3249.18 | 2951.56 | 1389.73 |
| Sodium | mg | 1700.59 | 1817.92 | 429.67 |
| Chloride | mg | 1952.46 | 2221.36 | ND |
| Magnesium | mg | 167.80 | 191.31 | 99.59 |
| Potassium | mg | 3868.58 | 3661.98 | 590 |
| Copper | mg | 3.536 | 3.019 | 0.001 |
| Iodine | mg | 0.129 | 0.219 | ND |
| Iron | mg | 91.96 | 95.20 | 2.0 |
| Manganese | mg | 1.663 | 1.612 | 0.000 |
| Selenium | mg | 0.152 | 0.155 | ND |
| Zinc | mg | 58.61 | 56.42 | 0.006 |
| ***Vitamins*** | | | | |
| Vit A | IU | 2973.59 | 3627.06 | 3200 |
| Vit D | IU | 722.04 | 693.10 | 371.94 |
| Vit E | IU | 15.117 | 24.381 | 2.596 |
| Vit K | mg | 0.105 | 0.168 | 0.090 |
| Biotin | mg | 0.047 | 0.039 | 0.015 |
| Choline | g | 0.211 | 0.219 | ND |
| Folacin | mg | 0.164 | 0.219 | 0.003 |
| Niacin | mg | 11.089 | 13.958 | 7.4 |
| Pantothenic Acid | mg | 4.953 | 7.276 | 3.95 |
| Riboflavin | mg | 1.651 | 1.574 | 1.75 |
| Thiamin | mg | 1.030 | 1.096 | 0.694 |
| Vit B6 | mg | 0.679 | 0.955 | ND |
| Vit B12 | µg | 3.525 | 6.244 | ND |
| ***Fatty Acid*** | | | | |
| 18:2 (Linoleic) | g | 9.461 | 9.469 | 8.504 |

| Table S2: Primers or probes were designed using primer express software | | | | | | |
| --- | --- | --- | --- | --- | --- | --- |
| **Gene** | **Primer sequence (5’- 3’)** | **Accession Number** |  | **Gene** | **Primer sequence (5’-3’)** | **Accession Number** |
| pActin 782 F  pActin 884 R | TCTTCCAGCCCTCCTTCTTG  GCGTAGAGGTCCTTCCTGATGT | XM_003357928 |  | pIL15-34F  pIL15-134R | TGCATCCAGTGCTACTTGTGTT TTAGGAAGACCTGCACTGATACAG | NM_214390 |
| pAMCFII-212F | ACCACACCCGGGATTCATC | NM_213876 |  | pIL16-1245F | CCATGTCACCATCTTGCACAA | NM_213751 |
| pAMCFII-312R | CCATTCTTCAGGGTGGCTATCA |  |  | pIL16-1345R | ACACCCTGTGAACCGTGATCA |  |
| pBMP2-754F | GATGAGCACAGCTGGTCACAA | NM_001195399 |  | pIL17A-123F | CCCTCAGCATGTAAGGGTCAAC | NM_001005729 |
| pBMP2-854R | TGTTTTGCTTGACGCTTTTCC |  |  | pIL17A-223R | GGAGAGTCCATGGTGAGGTGAA |  |
| pBMP4-1F | ATGATTCCTGGTAACCGAATGC | NM_001101031 |  | pIL17F-467F | TCCCCATCCAGCAAGAGTTC | NM_001924366 |
| pBMP4-101R | TTTTTCTTCCCCGTCTCAGGTA |  |  | pIL17F-568R | CACAGGTGCAACCAACAGTGA |  |
| pBMP6-1215F | CAGGAAGCACGAGCTCTACGT | NM_001168001 |  | pIL18-143F | GAAATCTGAACGACCAAGTCCTTT | NM_213997 |
| pBMP6-1315R | AGCACTCCCCGTCACAGTAATT |  |  | pIL18-243R | TACGGTCTGAGGTGCATTATCTGA |  |
| pCCL2-160F | GTCACCAGCAGCAAGTGTCCTA | NM_214214 |  | pIL21-179F | TGCCAGCTCCAGAAGATGTACA | NM_214415 |
| pCCL2-260R | ATGGAGTCCTGGACCCACTTCT |  |  | pIL21-279R | CTTTTCATTGTCTCCCGTATTTGC |  |
| pCCL4-68F | CAGCACCAATGGGCTCAGA | NM_213779 |  | pIL22-306F | AGTGCTGTTCCCCAACTCTGAT | XM_001926156 |
| pCCL4-169R | GGCTGCTGGTCTCATAGTAATCAG |  |  | pIL22-406R | CCTTAATACGGCATTGGCTTAGC |  |
| pCCL5-85F | GACACCACACCCTGCTGTTTTT | NM_001129946 |  | pIL23A-275F | CCTGCTTGCAAAGGATCCA | NM_001130236 |
| pCCL5-185R | ACTGCTGCCATGGAGCACTT |  |  | pIL23A-375R | CACAGAGCCATCAGGGTGTAGA |  |
| pCCL11-74F | CAGCTTCTGTCGCCACCAT | NM_001256774 |  | pIL27-311F | AACGACTCTGCTTCCTCTCCAT | NM_001007520 |
| pCCL11-174R | GGGACATTTGTTGGCAGTGACT |  |  | pIL27-411R | CAGCTGCATCCTCTCTGAACTG |  |
| pCCL20-86F | ACTTTGACTGCTGCCTCCGATA | NM_001024589 |  | pCSF3-22F | CTGCTGCTCTGGCACATTG | NM_213842 |
| pCCL20-186R | TGCATTGATGTCACAAGCTTCA |  |  | pCSF3-131R | TTCCTCACTTGCTCTAAGCACTT |  |
| pCCL21-173F | CCATCCCAGCTATCCTGTTCTC | NM_001005151 |  | pLTA-241F | AACACGGATCGTGCCTTCCT | NM_214453 |
| pCCL21-273R | GTCCAGATGGCGCATCAGTT |  |  | pLTA-341R | AAGACGACCTGGGAGTAGACAAA |  |
| pCCL22-138F | CCTGCGTGTGGTGAAGTATCA | NM_001256776 |  | pLTB-236F | AGGAGGCAGAAACAGATCTCA | NM_001185138 |
| pCCL22-257R | TTCTTCACCCAGGGCAGTCT |  |  | pLTB-345R | CAGAAACGCCTCTTCTTTCTTC |  |
| pCCL25-264F | GATCCTGGACAATCGGAATAAGA | NM_001025214 |  | pLIF-114F | CCCATGTCACAGCAACCTCAT | NM_214402 |
| pCCL25-364R | TTCCAGAGCTCAACTTCCTCACT |  |  | pLIF-215R | TCCCCCTGGGCTGTGTAGTA |  |
| pCCL27-55F | CCAGATCCTGGAGCAGCATT | NM_001003922 |  | pMIF-213F | GCAGAACCGTTCCTACAGCAA | NM_001077213 |
| pCCL27-155R | CGGATGATCTGCCTCAGTAGCT |  |  | pMIF-314R | GCCGCGTTCATGTCGTAGTA |  |
| pCXCL2 -151F | GGAATTCACCTCAAGAACATCCA | XM_003356973 |  | pVEGFA-130F | GACGTCTACCAGCGCAGCTACT | NM_214084 |
| pCXCL2 -252R | AGCTTCCTGACCATTCTTGAGAGT |  |  | pVEGFA-230R | ACACAGGACGGCTTGAAGATGT |  |
| pCXCL9-21F | CTTGCTTTTGGGTATCATCTTCCT | NM_001114289 |  | pFASL-355F | GCCAGCCAAAGGCATACAGA | NM_213806 |
| pCXCL9-121R | TCATCCTTTGGCTGGTGTTG |  |  | pFASL-455R | CCTGTTAAGTGGGCCACTTTTC |  |
| pCXCL10-40F | CTGACTCTGAGTGGAACTCAAGGA | NM_001008691 |  | pCD40L-356F | ATCCTCAAATTGCGGCACAT | NM_214126 |
| pCXCL10-140R | TTTTCTAAGGACCTCGGATTAACAG |  |  | pCD40L-456R | CAAGTTGGTGCTGAGGGTGTAG |  |
| pCXCL11-30F | GGCTGTCATATTTTGTGCTACA | NM_001128491 |  | pTNF-224F | AAGGACTCAGATCATCGTCTCAAA | NM_214022 |
| pCXCL11-130R | CTGCCACTTTCACTGCTTTTAC |  |  | pTNF-324R | GGCATACCCACTCTGCCATT |  |
| pCXCL12-68F | CGGTCAGCCTGAGCTACAGAT | NM_001009580 |  | pTNFSF10-325F | GGCATTCCTCACCTAGAAAGAGAA | NM_001024696 |
| pCXCL12-168R | GGCACAGTTCGGAGTGTTGAG |  |  | pTNFSF10-425R | TTGGAGCTTAGAGATGGAAATGTG |  |
| pIL1A-296F | CCATTGCCAATGACACAGAAGA | NM_214029 |  | pTNFSF13B-210F | GAGCAGCTCCATTCAAAGCAA | NM_001097498 |
| pIL1A-396R | GTGGTTGATGACCCTCATGAAGT |  |  | pTNFSF13B-310R | GCATGTCACTGTCTGCAATCAA |  |
| pIL-1B-290F | TTGAAGAAGAGCCCATCATCCT | NM_214055 |  | PTGFB1-320F | AGGTCACCCGCGTGCTAAT | NM_214015 |
| pIL-1B-390R | TTTGTGGTCTTTGTCCTGGAGTT |  |  | PTGFB1-420R | GAGCTCCGACGTGTTGAACAG |  |
| pIL5-33F | TCTTGGAGCTGCCTACGTTAGTG | NM_214205 |  | pTGFB2-284F | ACGAGGAATACTACGCCAAGGA | NM_003130499 |
| pIL5-133R | TCAGCAGAGTTCGATGAATGGA |  |  | pTGFB2-384R | CACTGAGCCAGAGGGTGTTGTA |  |
| pIL6-52F | CTGCTTCTGGTGATGGCTACTG | NM_214399 |  | pIFNA4-416F | CCATCCTGGCTGTGAGGAAATA | NM_001166319 |
| pIL6-152R | TCCGGAGAGGTGAAGAGCATT |  |  | pIFNA4-516R | CATGACTTCTGCCCTGATGATC |  |
| pIL8-29F | TGGCAGTTTTCCTGCTTTCTG | NM_213867 |  | pIFNA5-253F | CAGCAGACCTCCCAGCTCTT | NM_001164860 |
| pIL8-129R | AGGTGTGGAATGCGTATTTATGC |  |  | pIFNA5-353R | TCCCTGAGCTGCTGATCCA |  |
| pIL9-209F | AGGAGGGCCTATCGAAGATGAG | NM_001166043 |  | pLOC100518821-139F | AACATCAACCTGGACTCTGTGGAT | NM_003122707 |
| pIL9-309R | CTTGTTGTTCTTGAGGGCTTCA |  |  | pLOC100518821-239R | TAAGCTCGGAGGTTCTCTTGGA |  |
| pIL10-295F | GAGGATATCAAGGAGCACGTGAAC | NM_214041 |  | pLOC100152038-310F | CTGCACAGACTGACTGCTTTACAG | NM_001929161 |
| pIL10-395R | CTCTTGTTTTCACAGGGCAGAA |  |  | pLOC100152038-410R | TGGGACATGCAATGGATGTTAC |  |
| pIL12A-352F | ACAGTGGAGGCCTGCTTACCA | NM_213993 |  | pLOC100524265-69F | TGTTCTGGAGACCAATGACACAA | NM_003129101 |
| pIL12A-452R | CCAGAAGTCAGGCAATTTCCAT |  |  | pLOC100524265-174R | CCCAGGAGGCCAGATTTGA |  |
| pIL12B-610F | ATTGAGGTCGTGCTGGAAGCT | NM_214013 |  | pLOC100736831-342F | CGAGCCAGGGATGAAAGGT | NM_003480815 |
| pIL12B-710R | AGATTCTTGGGAGGGTCTGGTT |  |  | pLOC100736831-453R | TGCCAAGTGCAAGAACACATAGT |  |

| Table S3: Body weights at day 20. Statistical analyses of data was carried out by  mixed effect regression model. Data are presented as mean±SEM. | | | | | |
| --- | --- | --- | --- | --- | --- |
| **Females** | | | **Males** | | |
| **Milk** | **Sow** | **Soy** | **Milk** | **Sow** | **Soy** |
| N=6 | N=6 | N=6 | N=6 | N=6 | N=6 |
| 8.37±0.21^a^ | 7.80±0.26^a,b^ | 7.80±0.35^a,b^ | 7.72±0.27^a,b^ | 6.98±0.23^b^ | 7.97±0.44^a,b^ |
| Means sharing a letter are not significantly different at the 5% level.  *Bonferroni corrected *p*=0.0455 (Sow, Male) vs. (Milk, Female) | | | | | |

Figure S1: Chemokine cytokine data are shown as heat map. We have 12 animals/group and 6/gender. Data are normalized to unit variance. Generated in the R statistical environment.


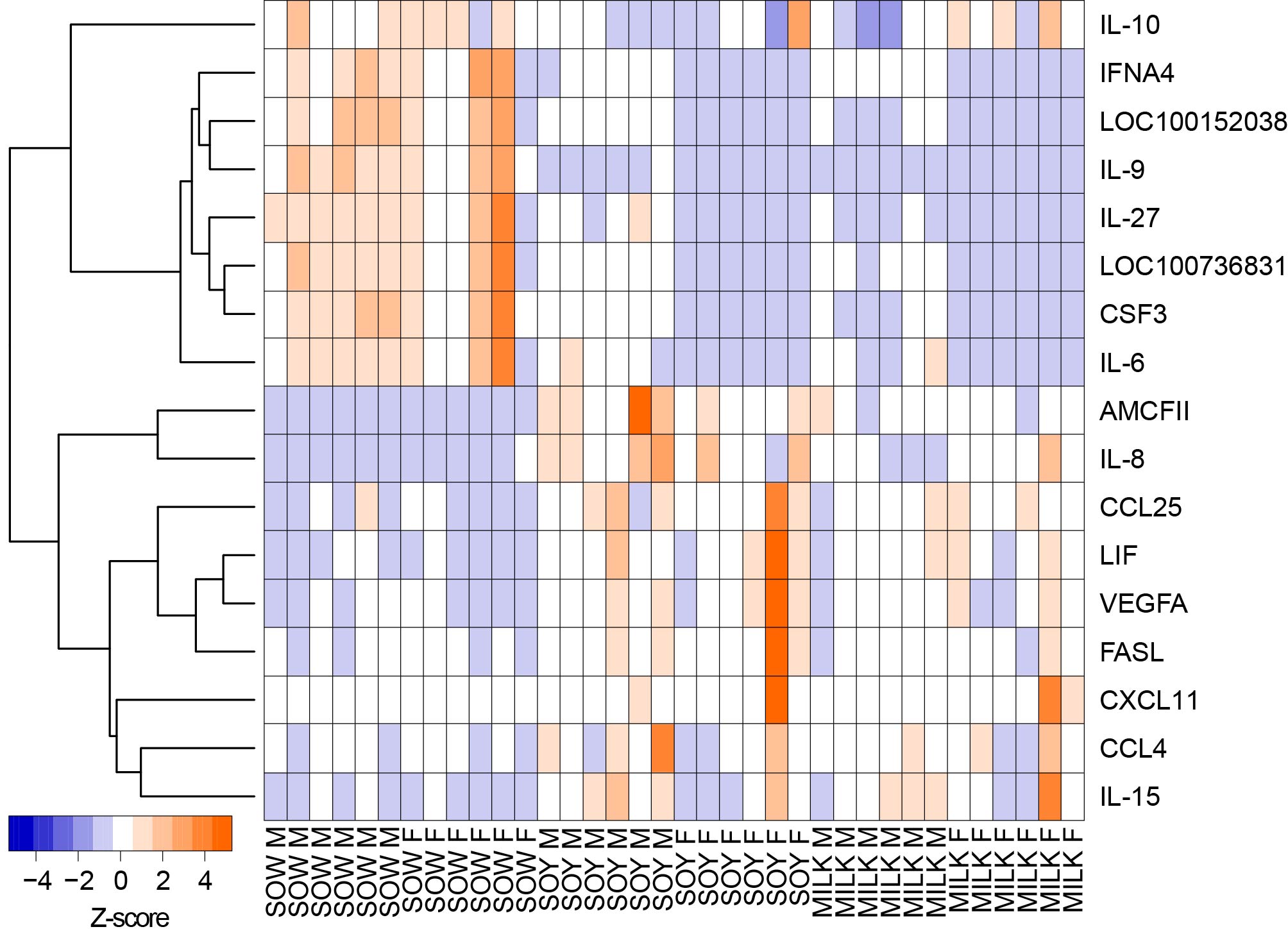

Supplement: Additional file 1: Table S1. — Diet composition of milk, soy and sow. Table S2. Primers or probes were designed using primer express software. Table S3. Body weights at day 20. Statistical analyses of data was carried out by mixed effect regression model. Data are presented as mean±SEM. Figure S1. Chemokine cytokine data are shown as heat map. We have 12 animals/group and 6/gender. Data are normalized to unit variance. Generated in the R statistical environment. (DOCX 314 kb) [file 12876_2016_456_MOESM1_ESM.docx]
